# Epidemiology of Epizootic Lymphangitis Among Carthorses in Ethiopia

**DOI:** 10.3389/fvets.2021.762937

**Published:** 2021-12-10

**Authors:** Musse G. Abdela, Sori Teshale, Mesfin M. Gobena, Aboma Zewde, Hawi Jaleta, Balako Gumi, Gobena Ameni

**Affiliations:** ^1^Aklilu Lemma Institute of Pathobiology, Addis Ababa University, Addis Ababa, Ethiopia; ^2^Department of Clinical Studies, College of Veterinary Medicine and Agriculture, Addis Ababa University, Bishoftu, Ethiopia; ^3^Department of Animal Sciences, University of Florida, Gainesville, FL, United States; ^4^The Ethiopian Public Health Institute (EPHI), Addis Ketema Sub-city, Addis Ababa, Ethiopia; ^5^School of Veterinary Medicine, College of Health Science, Wollega University, Nekemte, Ethiopia; ^6^Department of Veterinary Medicine, College of Food and Agriculture, United Arab Emirates University, Al Ain, United Arab Emirates

**Keywords:** epizootic lymphangitis, *Histoplasma capsulatum* variety *farciminosum*, epidemiology, carthorses, Ethiopia

## Abstract

Epizootic lymphangitis caused by *Histoplasma capsulatum* variety *farciminosum* is a debilitating disease incurring considerable economic losses and affecting the welfare of carthorses. Understanding of its epidemiology is important for devising effective prevention and control measures. A cross-sectional study was conducted on 4,162 carthorses in 17 towns in Ethiopia between October 2018 and June 2019. Clinical and microscopic examinations, fungal culturing, and polymerase chain reaction were used to undertake this study. The overall prevalence of epizootic lymphangitis was 16.67% (95% CI: 15.55–17.84) in carthorses. Epizootic lymphangitis was detected in carthorses found in 16 of the 17 towns included in the study. The highest prevalence was recorded at Kombolcha Town (33.33; 95% CI: 27.54–39.52) whereas the lowest was recorded at Debre Birhan Town (0.00; 95% CI: 0.00–1.27). The results of univariable firth logistic regression analysis showed that the difference between the prevalence of Kombolcha and the prevalences of all the other towns except Holota and Shashemene were statistically significant. Statistically significantly lower prevalence was observed in other towns. Classification of the cases into different clinical forms showed that 87.18, 4.33, and 0.58% were cutaneous, ocular, and respiratory forms respectively, while the remaining 7.93% (55/694; 95% CI: 6.03–10.19) were classified as mixed form. In terms of the severity of the disease, 28.67, 60.52, and 0.81% were mild, moderate, and severe cases, respectively. The majority of the lesions (43.95%) were observed in the skin followed by forelimbs (14.55%) and neck region (14.27%). Higher mean annual temperature, lower annual rainfall, and higher humidity of the study towns were statistically significantly associated with an increased risk of epizootic lymphangitis. In conclusion this study revealed widespread occurrence of epizootic lymphangitis in carthorses yet a heterogeneous prevalence between towns. The veterinary and livestock authorities should take this into account while devising disease control.

## Introduction

In Ethiopia, horses provide a multitude of services to both rural and urban communities, which are crucial for their livelihood. Horses still serve in several areas as the only available and affordable means of public and goods transportation (1). In certain parts horses provide draft power for preparation of croplands (1, 2). Despite their immense services, horses often suffer from a number of infectious diseases having considerable impacts on their welfare and livelihood of their owners. Epizootic lymphangitis is one of the infectious diseases posing huge socio-economic and welfare concerns on horses, specially carthorses. It is a contagious, chronic, debilitating disease caused by a thermally dimorphic fungus *Histoplasma capsulatum* variety *farciminosum* which occurs as a yeast form in host tissues and as a mycelial saprophytic form in the environment (3).

Although epizootic lymphangitis is endemic in several countries causing significant economic and welfare issues, it is an OIE non-listed disease. Based on the route of infection, epizootic lymphangitis can occur as cutaneous, respiratory, ocular, and asymptomatic forms (4). Cases involving a combination of all forms occur rarely. Transmission of epizootic lymphangitis mostly occurs through contacts with infectious cases, but the role of inanimate objects (harness and other implements) and fly vectors via broken skin cannot be ruled out (4, 5).

Epizootic lymphangitis was widespread in Europe during the late nineteenth and twentieth centuries and was eradicated through regular surveillance and slaughter programs (6). Currently it is common in many African countries, the Middle East, Russia, and Asia with tropical and subtropical climates (2, 5, 7, 8).

In Ethiopia, studies showed that it is a widespread disease affecting 18.8–26.2% of carthorses (7, 9) and 21% of cart mules (2). Previous surveys conducted a decade ago reported no cases of epizootic lymphangitis in areas characterized by cool temperature and higher elevation (2, 7, 9). Yet recent personal observations indicate that epizootic lymphangitis can be observed in carthorses in some towns with cool temperature and high-altitude such as Dessie (northern Ethiopia), Holeta, and Sebeta (central Ethiopia). To better characterize the situation in these areas and to update and identify risk factors associated with epizootic lymphangitis distribution in Ethiopia, we investigated the epizootic lymphangitis prevalence in carthorses in 17 selected towns of Ethiopia. This will help design tailored intervention strategies aimed at reducing its occurrence.

## Materials and Methods

### Study Area

**Figure 1:**
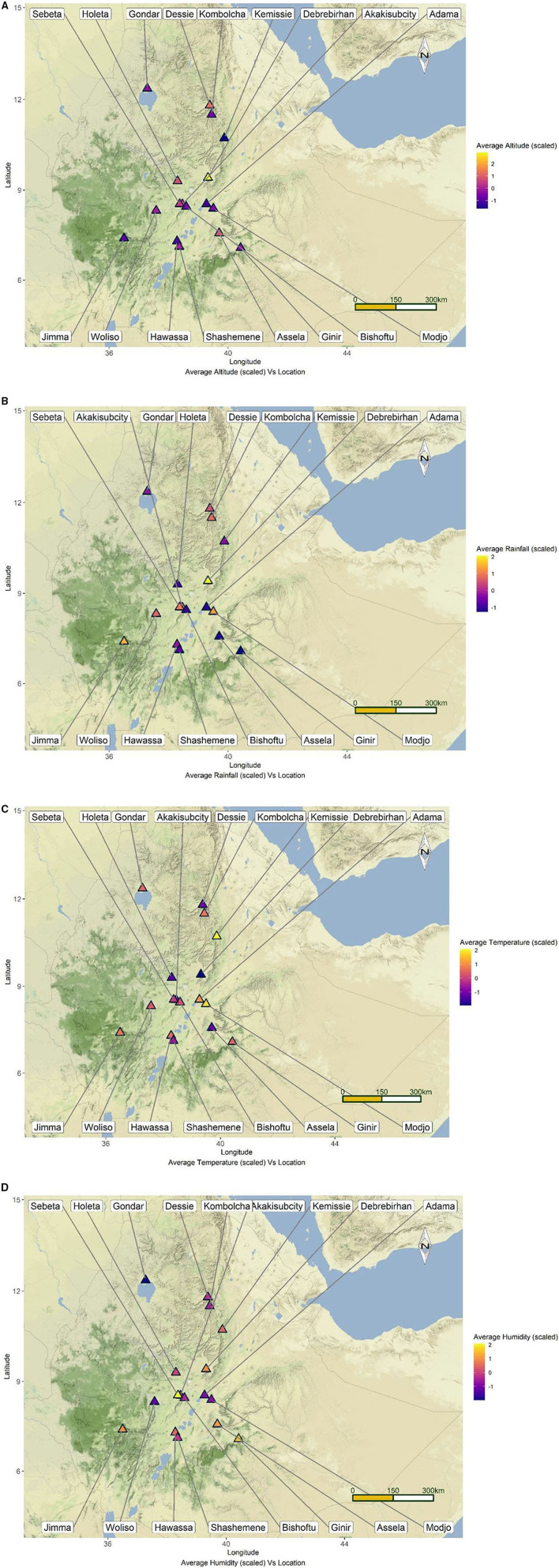
Map of Ethiopia depicting the locations of the study towns along with their mean altitude **(A)**, rainfall **(B)**, temperature **(C)**, and humidity **(D)**.

The study was conducted in 17 towns selected from Amhara and Oromia National Regional States of Ethiopia (Figure 1). Amhara and Oromia Regional States are the two largest states in terms of area coverage, human population, and natural resources including livestock. Together Oromia and Amhara regions have a total of 1,572,532 horses which is 78.6% of the total horse population of the country (6). The climatic and ecological features these regions are very conducive for agriculture and livestock production. These two regions were, therefore, selected purposively. The remaining regional states are either predominantly pastoral or situated in the tse tse fly belt where horses are rare. The towns were selected purposively based on the number of carthorses present, the climatic conditions, and their accessibility to transportation to avoid spoilage of samples. The mean annual temperature of the study towns ranges from 21.1 to 31.2°C; the mean annual rainfall ranges from 83.8 to 167.5 mm; the mean annual humidity ranges from 56 to 83%, and the average altitude ranges from 1,438 to 3,206 m above sea level (Table 1). The towns are featured by fast urbanization where the demand for transportation is escalating. To this end the number carthorses providing transportation services is increasing as well.

**Table 1:** The mean annual temperature, rainfall, humidity and average altitude of the study towns.

**Town**	**Temperature (**°**C)**	**Rainfall (mm)**	**Humidity (%)**	**Altitude (m asl)**
Adama	28.5	88.0	59	1,622
Akaki	23.0	131.8	62	2,354
Asella	23.1	86.5	75	2,413
Bishoftu	26.5	90.0	63	1,900
Debrebirhan	21.1	167.5	74	3,206
Dessie	23.5	128.7	64	2,553
Gindhir	27.1	83.8	79	1,941
Gondar	27.2	110.0	50	1,973
Hawassa	27.3	111.0	70	1,694
Holota	22.4	93.0	66	2,391
Jimma	27.8	152.4	74	1,718
Kemisse	31.2	108.0	70	1,438
Kombolcha	27.7	135.9	62	1,857
Modjo	30.5	147.2	60	1,870
Sebeta	25.7	140.0	83	2,220
Shashemene	24.5	97.0	65	2,080
Weliso	26.8	134.8	56	2,058

### Study Population and Design

A cross-sectional study was conducted from October 2018 to May 2020 to investigate the epidemiology of epizootic lymphangitis in carthorses in Ethiopia. The study population comprises carthorses found in the selected towns. The total number of carthorses in each town was obtained from the municipalities of each town as it is mandatory for owners to register for issues related to administration and tax. There are 4–5 carthorse stations in each town and these stations were used as sampling sites. The carthorses and their drivers wait for customers at the stations where they pick passengers or goods. There are a total of 4,380 registered carthorses in the 17 towns. Each cart owner has two horses that pull the same cart. One of the horses is used during the morning hours whereas the other one is used in the afternoon while the cart remains the same. For this reason, each carthorse station was visited twice a day, in the morning and in the afternoon, in order to examine and sample all horses. Horses are used singly to pull carts and no pairing was observed. The horses are stabled in the same enclosure at night. During this study a total of 4,162 carthorses were sampled (nearly 95% of the total carthorses in the selected towns). The remaining 5% were not included due to unwillingness of the owners.

### Clinical Examination of Horses

All carthorses found in the stations were clinically examined for the presence of any lesions suggestive of epizootic lymphangitis by inspection and palpation, particularly for the presence of nodules and/or ulcers. Great emphasis was given to nostrils and eyes, the skin, and lymphatics during physical examination of the horses. Based on the clinical examination the horses were classified as healthy (no detectable lesion), clinically positive cases (having mild, moderate, or severe lesion scores) based on the the grading system described by Mideksa et al. (10), and confirmed cases (horses with a positive smear). The owners and drivers of each carthorse were asked if they had seen any visible epizootic lymphangitis lesions on their carthorses.

### Sample Collection

Samples were collected from all horses suspected to have lesions of epizootic lymphangitis. Pus samples were aspirated aseptically by sterile needles and syringes from un-ruptured nodules. The nodules were washed using water and soap, shaved with sterile scalpel blade, and disinfected with 70% alcohol. For nodular lesions observed on skin and lymphatics, sterile syringe and needles were used to aspirate the pus samples. Sterile 10 cm long swab was used to collect pus samples from the ocular and respiratory forms of epizootic lymphangitis. The samples collected were immediately transferred into a sterile universal bottle containing saline solution and kept cool using an icebox. The collection was done after obtaining permission from owners of the carthorses under the supervision of local administration. The bottles were labeled and transported to Veterinary Microbiology Laboratory, Akililu Lemma Institute of Pathobiology, Addis Ababa University for laboratory examination. The samples were stored refrigerated at 4°C and processed within 24–48 h of submission. When sample processing was not possible within 48 h, they were frozen at −80°C.

Geo-referenced data were collected during sample collection from each town using Android version 4.4 phones, which captured the altitudes of the towns and the Geographical Positioning System device was used to identify the latitude and longitude of the towns. Climatic data such as the mean annual temperature, the mean annual rainfall, and the mean annual humidity of each town were obtained from National Meteorological Agency of Ethiopia.

### Microscopic Examination

For microscopic examination smear was prepared from the pus and swab samples on glass slide in the field prior to transfer into sterile saline, fixed with methanol, and kept on the slide box. The smears were stained with Wright-Giemsa stain and examined under microscope for the typical yeast form of *Histoplasma capsulatum* variety *farciminosum* which appears as ovoid to globose structures occurring singly or in group either inside cells or in extracellular space (4). The horses with positive smears were considered as confirmed positives and were used to compute prevalence of epizootic lymphangitis in this study.

### Isolation of *Histoplasma capsulatum* Variety *farciminosum*

Primary isolation of *Histoplasma capsulatum* variety *farciminosum* was done on Brain Heart Infusion Aagar (BHIA) with 5% horse blood and 0.005% chloramphenicol. Chloramphenicol (0.5 g/L) was added to the media to avoid the growth of bacterial contaminants. The inoculated media were incubated at 26°C and 5% CO_2_ for about 8–12 weeks (4). The growth of the mycelial form of *Histoplasma capsulatum* variety *farciminosum* was evident when dry, gray-white, granular wrinkled colonies were observed. Typical colonies were collected and stained with Gram's stain for demonstration of the morphologic features of *Histoplasma capsulatum* variety *farciminosum*. From the primary cultures, typical colonies were further sub-cultured onto Sabouraud dextrose agar (SDA, Oxoid) enriched with 2.5% glycerol and 0.005% chloramphenicol. The isolates with typical colony appearance and morphologic features of *Histoplasma capsulatum* variety *farciminosum* were subjected to nested polymerase chain reaction (PCR) for confirmation of their identity.

### Molecular Identification of *Histoplasma capsulatum* Variety *farciminosum*

#### Extraction of DNA

The modified sodium dodecyl sulfate (SDS)-cetyl-trimethylammonium bromide (CTAB)-chloroform-isoamyl alcohol method was used for the extraction of DNA (11). Briefly, 200 mg of mycelial growth was taken and transferred to 2 mL Eppendorf tube. Five hundred microliter of SDS-CTAB-chloroform-isoamyl alcohol extraction buffer (250 mM Tris-HCl [pH 8.0], 20 m MEDTA [pH 8.0], 200 M NaCl, 10% CTAB, and 0.15% SDS) was added and vortexed. The mixture was boiled at 50°C for 10 mins and centrifuged at 10,000 g for 10 mins. The supernatant was aspirated carefully and mixed with one volume of chloroform: isoamyl alcohol (23:2) for 1 min and centrifuged at 10,000 g for 5 mins. The aqueous phase was collected and mixed with one volume of ice-cold isopropanol, and the tubes were turned upside down for 1 min to precipitate the DNA. The tubes were centrifuged at 10,000 g for 2 mins to recover the pellet and washed with 500 mL of absolute ethanol and then centrifuged at 10,000 g for 1 min. The pellet was air dried, and the DNA was resuspended in 200 mL deionized or TE buffer. The DNA was either used for amplification or stored at −20°C until analyzed.

### Amplification and Visualization

A nested PCR amplifying about 514 bp of DNA was used as described by Jiang et al. (12) and Scantlebury et al. (13) using primers designated P3, 2R8, F5, and 2R5 supplied by Eurofins Genetics, Germany. DNA of *H. capsulatum* var. *farciminosum* reference strain CBS 539.84 was used as positive control whereas extracts of *Saccharomyces cerevisiae* DNA were used as negative control. Each PCR reaction mixture contained 50 ng/mL of template DNA and 10 pmol of PCR primers P3 and 2R8 added to BioMix red (Bioline Reagents Limited, UK) in a 25 mL reaction volume, as follows: 12 mL BioMix red, 2 mL forward primer, and 2 mL reverse primer, 8 mL water, and 1 mL of DNA template (50 ng/μL). The first round of PCR was run using the P3 (forward primer: 50-CGGAAGGATCATTACCACGCCG-30) and 2R8 (reverse primer: 50-CAGCGGGTATCCCTACCTGATC-30) in the thermal cycler (Eppendorf Master cycler) with denaturation at 94°C for 10 mins followed by 35 cycles of the succeeding steps of denaturation at 94°C for 1 min, annealing at 49°C for 1 min and extension at 72°C for 1 min and a final extension period of 72°C of 7 mins. A 1-in-10 (v/v) dilution of the product from the first reaction was added to fresh master mix and amplified using forward primer: F5 (50-CTACCCGGCCACCCTTGTCTAC-30) and reverse primer: 2R5 (50- CCTACCTGATCCAGTCAACC-30). The reaction cycles for the second round of PCR were the same as the first round, except that the annealing temperature was raised to 55°C for 1 min. The PCR products were visualized by gel electrophoresis using 2% agarose (Sigma Chemical Co, St. Louis, MO) dissolved in Tris-borate-EDTA buffer (0.1 M Tris, 0.09 M boric acid, and 0.001 M EDTA [pH 8.4]), allowing it to solidify and finally staining with ethidium bromide (3 mL per 100 mL agarose). The electrophoresis was conducted at 90 V for 50 mins by mixing 5 mL of each PCR product with l mL of loading dye in each well. The bands were visualized with a UV transilluminator.

### Data Analysis

Data were checked, coded, and entered into Microsoft Excel 2010 (Microsoft Corp., Redmond, USA) and analyzed using Stata version 14 (StataCorp, 4905 Lakeway Drive, College Station, Texas 77845 USA). Prevalence was calculated by dividing the number of smear positive carthorses by a total number of carthorses examined then multiplied by 100 and 95% CI was constructed. Multivariable logistic regression was used compute associations between the prevalence of epizootic lymphangitis and climatic factors such as altitude, annual temperature, annual humidity, and annual rainfall of the study towns (National Meteorology Service). The association between the prevalence and the study towns was assessed by univariable firth logistic regression analysis. A difference was taken as significant at a *p*-value ≤ 0.05.

## Results

### Prevalence of Epizootic Lymphangitis

A total of 4,380 carthorses were encountered, of which 4,162 examined clinically for epizootic lymphangitis. Overall 577 horses had showed clinical signs of which 694 gave smear positive results yielding a prevalence of 16.67% (95% CI: 15.55–17.84). Epizootic lymphangitis was detected in carthorses found in 16 of the 17 towns included in the study. The highest prevalence was recorded at Kombolcha Town (33.33; 95% CI: 27.54–39.52) followed by Shashemene Town (24.25; 95% CI: 20.13–28.76) whereas the lowest was recorded at Debre Birhan Town which was 0.00% (95% CI: 0.00–1.27). The results showing the prevalence of epizootic lymphangitis per town is presented in Table 2. The results of univariable firth logistic regression analysis showed that the differences between the prevalence of Kombolcha and the prevalences of all the other towns except Holota and Shashemene were statistically significant (Table 3).

**Table 2:** Prevalence of Epizootic Lymphangitis in carthorses per town.

**Town**	**N^**0**^ examined**	**N^**0**^ positive**	**Prevalence**	**95 % CI**
Adama	150	15	10.00	5.71–15.96
Akaki	80	12	15.00	7.99–24.74
Assela	360	43	11.94	8.78–15.75
Bishoftu	35	75	21.43	17.25–26.10
Debrebirhan	234	0	0.00	0.00–1.27
Dessie	150	6	4.00	1.48–8.51
Gindhir	120	18	15.00	9.14–22.67
Gondar	384	34	8.85	6.21–12.15
Hawassa	410	73	17.81	14.23–21.86
Holota	307	91	22.64	24.59–35.09
Jimma	302	59	19.54	15.22–24.46
Kemisse	112	18	16.07	9.81–24.21
Kombolcha	252	84	33.33	27.54–39.52
Mojo	200	8	4.00	1.75–7.73
Sebeta	150	31	20.67	14.49–28.03
Shashemene	400	97	24.25	20.13–28.76
Weliso	201	30	14.93	10.31–20.62
**Total**	**4,162**	**694**	**16.67**	**15.55–17.84**

**Table 3:** Results of firth logistic regression analysis comparing the prevalence of Epizootic Lymphangitis among the towns studied.

**Location**	**OR**	**SE**	**Z**	***P*-value**	**95 % CI**
Adama	0.23	0.07	−4.95	0.000	0.13–0.41
Akaki	0.36	0.12	−3.03	0.002	0.19–0.69
Assela	0.27	0.06	−0.62	0.000	0.18–0.41
Bishoftu	0.54	0.10	−3.28	0.001	0.38–0.78
Debrebirhan	0.01	0.01	−3.84	0.000	0.001–0.69
Dessie	0.1	0.04	−5.72	0.000	0.04–0.20
Gindhir	0.36	0.10	−3.60	0.000	0.21–0.63
Gondar	0.19	0.04	−7.33	0.000	0.13–0.30
Hawassa	0.43	0.08	−4.53	0.000	0.30–0.62
Holota	0.84	0.15	−0.97	0.33	0.59–1.19
Jimma	0.48	0.09	−3.68	0.000	0.33–0.71
Kemisse	0.39	0.11	−3.30	0.001	0.22–0.68
Mojo	0.09	0.03	−6.49	0.000	0.04–0.18
Sebeta	0.52	0.13	−2.70	0.007	0.33–0.84
Shashemene	0.94	1.12	−0.55	0.11	0.50–1.01
Weliso	0.35	0.08	−4.39	0.000	0.22–0.56
Constant	0.50	0.07	−5.13	0.000	0.39–0.66

### Diagnosis of Epizootic Lymphangitis

**Figure 2:**
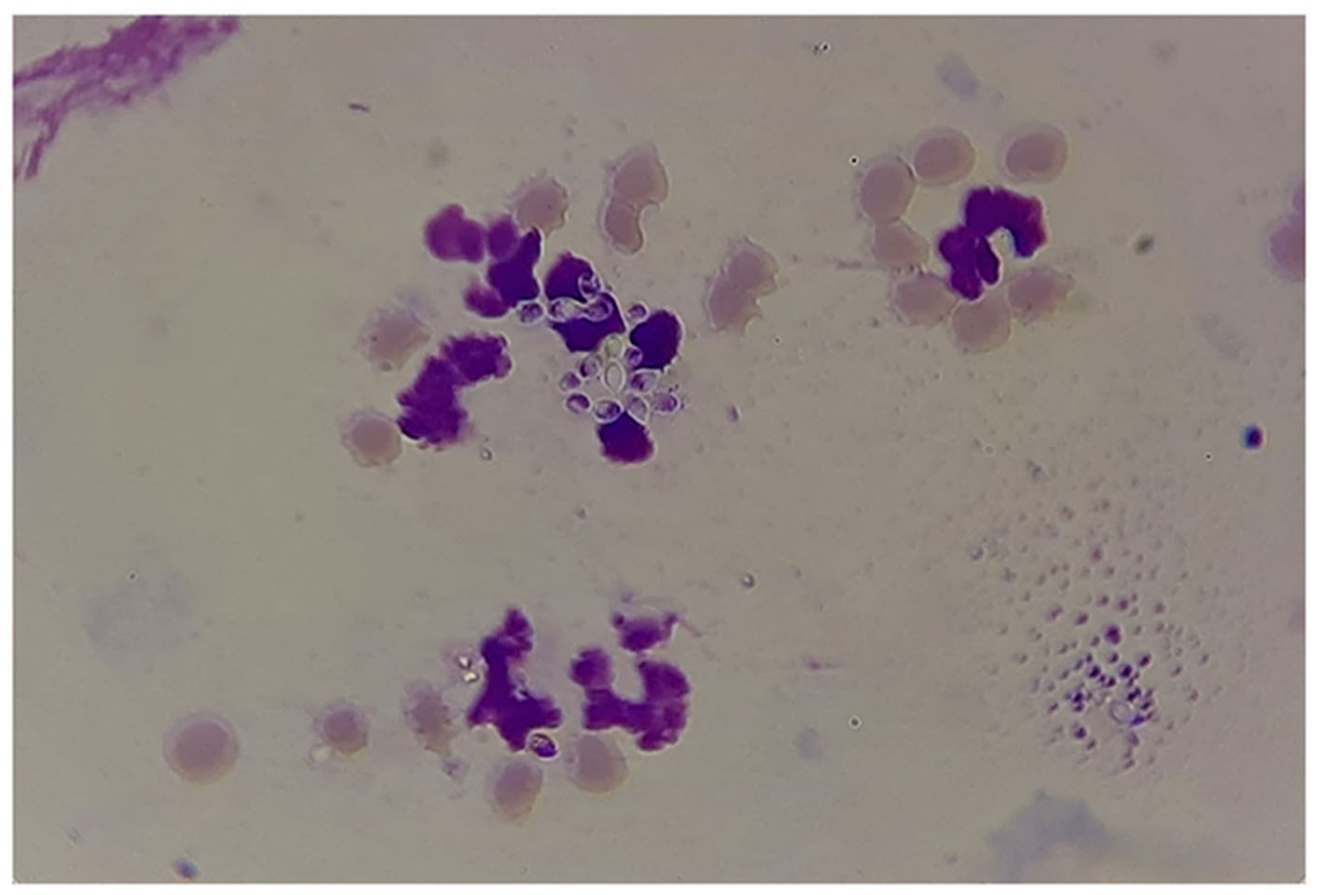
Results of microscopic examination of morphological appearance of the yeast form of *Histoplasma capsulatum* variety *farciminosum* (arrows).

**Figure 3:**
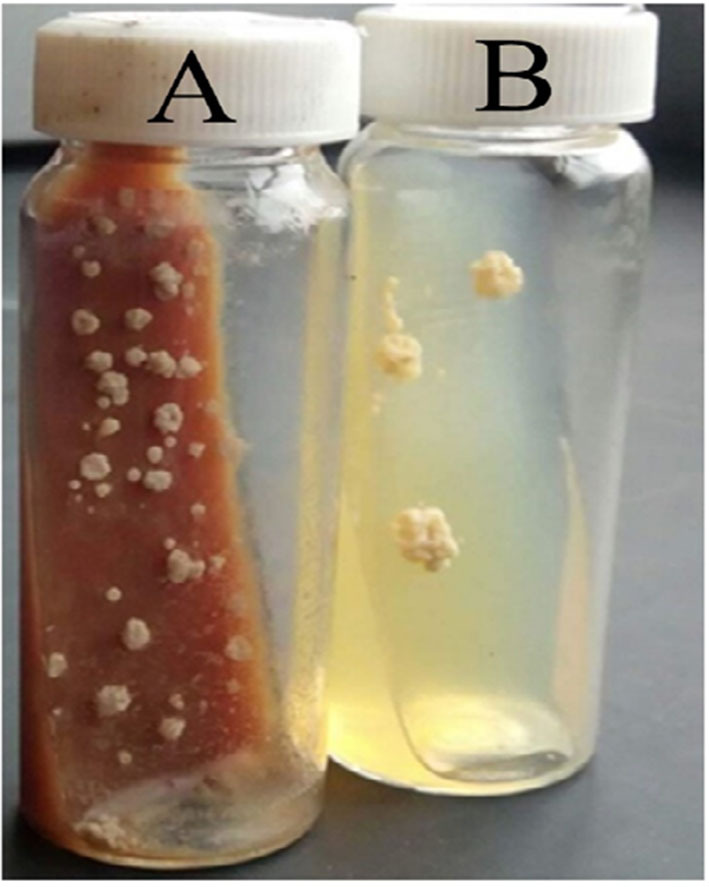
Cultural appearances of *Histoplasma capsulatum* variety *farciminosum*. **(A)** 52 days old, primary culture of mycelial colonies on Brain Heart Infusion Aagar (with 5% horse blood and 0.005% chloramphenicol); **(B)** 48 days old sub-culture of the mycelial form on Sabouraud Dextrose Agar (with 2.5% glycerol and 0.005% chloramphenicol).

The results of microscopic examination of smears prepared from fresh pus samples and swab samples revealed the occurrence of double contorted yeast cells featured by gram-positive, ovoid aggregates surrounded by a halo (Figure 2). Brown, dry, wrinkled colonies were cultured after 2 months of incubation at 26°C (Figures 3A,B). The colonies sub-cultured onto SDA supplemented with 2.5% glycerol revealed the growth of whitish to whitish-brown, dry, convoluted colonies, which revealed branching hyphae, chlamydospore, and macroconidia in Lactophenol cotton blue wet mounts examined under oil immersion.

**Figure 4:**
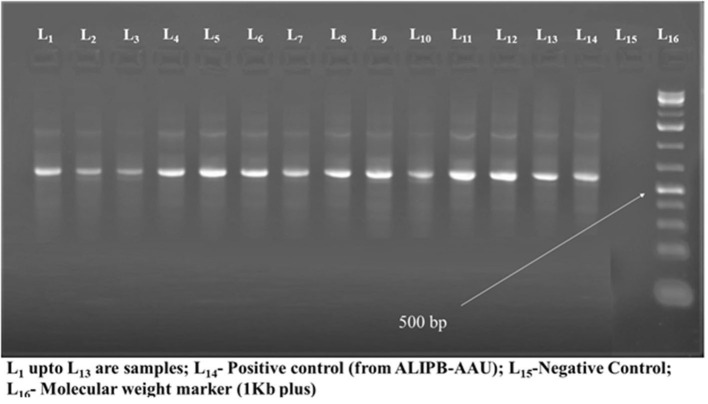
The results of molecular diagnosis of *Histoplasma capsulatum* variety *farciminosum* using PCR.

Thirteen typical colonies from sub-culture were further tested by using conventional PCR using primers designated ITS1 and ITS4. The occurrence of DNA fragment of about 514 bp revealing the involvement of *Histoplasma capsulatum* variety *farciminosum* was observed in those samples tested (Figure 4). Since the samples were collected from clinically affected horses the results of morphological, cultural, and molecular analysis were used to confirm the identity of *Histoplasma capsulatum* variety *farciminosum*.

Almost all of the carthorses with clinical signs showed one or more freely movable or firm nodules as well as ulcers on different parts of their bodies. Classification of the cases into different clinical forms showed that 87.18% (95% CI: 84.46–89.57), 4.33% (95% CI: 2.94–6.11), 58% (4/694; 95% CI: 0.16–1.47) were cutaneous, ocular and respiratory forms, respectively while the remaining 7.93% (55/694; 95% CI: 6.03–10.19) were classified as mixed form. In terms of the severity of the disease, 28.67% (95% CI: 25.34–32.19), 60.52% (95% CI: 56.77–64.18), and 0.81% (95% CI: 8.59–13.36) were mild, moderate, and severe cases, respectively. The majority of the lesions (43.95%) were observed in lymphatics along the limbs followed by forelimbs (14.55%) and neck region (14.27%). The distribution of the lesions is presented in Table 4.

**Table 4:** Distribution of the lesions of Epizootic Lymphangitis in carthorses in the study areas.

**Body part**	**N^**0**^ horses**	**Percent**	**95 % CI**
Lymphatic lines of the Limbs alone	305	43.95	40.22–47.73
Neck region alone	99	14.27	11.75–17.09
Neck and Forelimbs	101	14.55	12.01–17.39
Perineum	10	1.44	0.69–2.64
Sternal and ventral region	55	7.93	6.03–10.19
Muzzle and Head	35	5.04	3.54–6.94
Inguinal area	25	3.60	2.35–5.27
All over the body	64	9.22	7.17–11.62

### Effects of Climatic Factors and Altitude on Epizootic Lymphangitis

The results of multivariable logistic regression analysis showed the mean annual temperature, average humidity, and mean annual rainfall of the study towns were statistically significantly associated with occurrence of epizootic lymphangitis. As altitude and temperature were strongly correlated (*r*^2^ = 0.79), we built a model including only temperature, rainfall, and humidity, and then present the model with these variables. The odd of being infected with epizootic lymphangitis was 1.63 times higher in towns with mean annual temperature ranging from 27.1 to 31.2°C than those with lower temperature. Similarly the odd of infection was 2.54 times higher in towns that had average humidity in the range of 61–83% compared those with lower humidity. Towns receiving mean annual rainfall lower than 100 mm had higher odds of infection than those receiving higher rainfall. However, altitude was not associated with occurrence of epizootic lymphangitis (Table 5).

**Table 5:** The results of logistic regression analysis on the effects of climatic factors on the occurrence of epizootic lymphangitis in carthorses in Ethiopia.

**Variable**	**OR**	**SE**	**Z**	***P*-value**	**95 % CI**	
Temperature (°C)						
21.1–27.0	Ref					
27.1–31.2	1.63	0.17	4.65	**0.000**	1.33	2.01
Rainfall (mm)						
83.8–100	Ref					Ref
101–167.5	0.68	0.07	−3.84	**0.000**	0.56	0.83
Humidity (%)						
56–60	Ref					
61–83	2.54	0.39	5.97	**0.000**	1.87	3.44
Constant	0.09	0.02	−14.31	0.000	0.07	0.14

*The bold values show the occurrence statistically highly significant association*.

## Discussion

Horses remain invaluable animals serving the community in pack and saddle transport. However, their welfare and optimum service is hampered by diseases such as epizootic lymphangitis. In this study, the occurrence of epizootic lymphangitis was investigated in carthorse in 17 towns of Ethiopia to estimate its prevalence and assess the effects of climatic factors on its occurrence. The results revealed that epizootic lymphangitis occurs in 16 of the 17 towns investigated with overall prevalence of 16.67%. This has significant implication for the carthorse owners and the public, who use cart transportation. This observation is in agreement with previous report of Ameni (7) which reported a prevalence of 18.76% in 19,082 carthorses in 28 towns of Ethiopia. Although the sample size and area coverage are limited in our study the results confirm to the previous study (7) suggesting the widespread occurrence of epizootic lymphangitis in carthorses throughout Ethiopia. On the other hand the prevalence observed in this study is lower than the reports of Hadush et al. (14) from northern Ethiopia (44%). Our observation is higher than the reports of Wilson (15) from Sudan (17–58 cases per year) and that of Abdullahi et al. (16) from Nigeria (5.6%). In addition to the overall prevalence, the highest and lowest prevalence observed in this study were similar to those reported earlier by Ameni (7). However, variations in prevalence were observed in some of the towns included in the study. Previous studies reported high prevalence in 11 towns including Mojo, Jimma, Bishoftu (7), and Weliso (24.9%) reported by Asfaw et al. (17) where low to moderate prevalence is recorded in this study. The lower prevalence observed in Mojo and Bishoftu in this study could be attributed to intervention made by the Society for Protection of Animals Abroad (SPANA) through provision of training and harnesses that minimize wounds to the horses. In contrast, higher prevalence was recorded at Kombolcha, Shashemene, and Holota towns, where either low or no cases of epizootic lymphangitis were reported by previous researchers (7).

Interestingly, in both this study and the previous ones a large number of cases of epizootic lymphangitis were recorded in towns receiving mean annual rainfall ranging from 80 to 410 mm, suggesting the role of climatic factors on the survival and spread of the fungus. In this study mean annual temperature, mean annual rainfall, and humidity are statistically associated with the occurrence of epizootic lymphangitis. Those towns having higher average temperature (27.1–31.2°C), average rainfall ranging from 83.8 to 100 mm, and average humidity of 61–83% had higher odds of occurrence of epizootic lymphangitis in horses. Altitude was strongly correlated with temperature; towns located at lower altitudes exhibited higher temperatures and were significantly more at risk of epizootic lymphangitis. The significant association between climatic factors and the risk of epizootic lymphangitis could be due to the survival of the mycelial stage of *Histoplasma capsulatum* variety *farciminosum* in the environment and on its transmission among horses. In agreement with our observation other authors documented that temperature up 29°C and humidity of 67–87% are favorable for the survival of the mycelial form of the fungus. Rainfall and or humidity can cause maceration of the skin which can easily be traumatized and allow entrance of the fungus into the body of horses (18). Here a higher humidity but a lower rainfall was associated with an increased risk of epizootic lymphangitis. A finer analysis would be necessary to disentangle the combined effects of rainfall, humidity, and temperature and to determine whether higher rainfall in areas having higher temperatures leads to increased humidity, which in turn could stress the animals and predispose them to infections. Previous studies revealed that the occurrence of epizootic lymphangitis is common in areas characterized by warm and humid conditions with altitude ranging from 1,500 to 2,300 m above sea level (2, 7, 19). In addition, climatic factors such as rainfall also have effects on the skin of horses. Higher rainfall in areas having higher temperature also increases humidity, which stress the animals and predispose them to infections including *Histoplasma capsulatum* variety *farciminosum*.

The majority (87.18%) of epizootic lymphangitis cases observed were cutaneous forms followed by ocular and pulmonary forms, which is in agreement with the findings of Ameni et al. (7) and Asfaw et al. (17). The frequent occurrence of the lesions over the limbs, neck, and the chest area could be due the direct exposure of the skin over these areas to the effects of harnesses, which causes trauma that favors the entrance of the *Histoplasma capsulatum* variety *farciminosum* into the tissues. In addition, the lateral aspects of the body of horses and the limbs are often exposed to chaffing and nails and harnesses that have rigid and rough edges, which create wounds on the skin. The anatomical distribution of epizootic lymphangitis lesions observed in this study are in agreement with the previous reports in horses and mules (2, 7, 17).

In conclusion, this study revealed the widespread occurrence of epizootic lymphangitis in carthorses in 16 of the 17 towns included in this study ranging from 0.00% at Debre Birhan to 33.33% at Kombolcha with an overall prevalence of 16.67%. Statistically significant difference was observed in the prevalence of epizootic lymphangitis and the localities. Climatic factors such as temperature, rainfall, and humidity are important risk factors affecting its occurrence in carthorses in the study towns. The veterinary and livestock authorities should take this into account and devise control strategies to minimize the impacts of the disease and its spread.

## Data Availability Statement

The original contributions presented in the study are included in the article/supplementary material, further inquiries can be directed to the corresponding author.

## Ethics Statement

The animal study was reviewed and approved by Institutional Review Board of Aklilu Lemma Institute of Pathobiology, Addis Ababa University.

## Author Contributions

MA, ST, AZ, and HJ: participated in the collection of field data. GA and ST: developed the proposal and solicited fund. ST, MA, and MG: drafted the paper. GA, BG, MG, and HJ: edited the drafted paper. MA, MG, and AZ: performed laboratory analysis. BG: provided laboratory reagents. All authors contributed to the article and approved the submitted version.

## Funding

This work was supported by small amount of grant provided by the Addis Ababa University through its sixth Thematic Research Fund (2017/2018 academic year). We declare that the funding body did not contribute to the execution of any part of the study, interpretation of the results, and write up of this article.

## Conflict of Interest

The authors declare that the research was conducted in the absence of any commercial or financial relationships that could be construed as a potential conflict of interest.

## Publisher's Note

All claims expressed in this article are solely those of the authors and do not necessarily represent those of their affiliated organizations, or those of the publisher, the editors and the reviewers. Any product that may be evaluated in this article, or claim that may be made by its manufacturer, is not guaranteed or endorsed by the publisher.
